# A hybrid correcting method considering heterozygous variations by a comprehensive probabilistic model

**DOI:** 10.1186/s12864-020-07008-9

**Published:** 2020-11-18

**Authors:** Jiaqi Liu, Jiayin Wang, Xiao Xiao, Xin Lai, Daocheng Dai, Xuanping Zhang, Xiaoyan Zhu, Zhongmeng Zhao, Juan Wang, Zhimin Li

**Affiliations:** 1 School of Computer Science and Technology, Xi’an Jiaotong University, Xi’an, 710048 China; 2 Shaanxi Engineering Research Center of Medical and Health Big Data, School of Computer Science and Technology, Xi’an Jiaotong University, Xi’an, 710048 China; 3 School of Public Policy and Administration, Xi’an Jiaotong University, Xi’an, 710048 China; 4 Annoroad Gene Institute, Beijing, 100176 China

**Keywords:** Sequencing analysis, PacBio sequencing, Sequencing error, Error correction method, Hybrid correction method, Heterozygous variant, Probabilistic model

## Abstract

**Background:**

The emergence of the third generation sequencing technology, featuring longer read lengths, has demonstrated great advancement compared to the next generation sequencing technology and greatly promoted the biological research. However, the third generation sequencing data has a high level of the sequencing error rates, which inevitably affects the downstream analysis. Although the issue of sequencing error has been improving these years, large amounts of data were produced at high sequencing errors, and huge waste will be caused if they are discarded. Thus, the error correction for the third generation sequencing data is especially important. The existing error correction methods have poor performances at heterozygous sites, which are ubiquitous in diploid and polyploidy organisms. Therefore, it is a lack of error correction algorithms for the heterozygous loci, especially at low coverages.

**Results:**

In this article, we propose a error correction method, named *QIHC*. *QIHC* is a hybrid correction method, which needs both the next generation and third generation sequencing data. *QIHC* greatly enhances the sensitivity of identifying the heterozygous sites from sequencing errors, which leads to a high accuracy on error correction. To achieve this, *QIHC* established a set of probabilistic models based on Bayesian classifier, to estimate the heterozygosity of a site and makes a judgment by calculating the posterior probabilities. The proposed method is consisted of three modules, which respectively generates a pseudo reference sequence, obtains the read alignments, estimates the heterozygosity the sites and corrects the read harboring them. The last module is the core module of *QIHC*, which is designed to fit for the calculations of multiple cases at a heterozygous site. The other two modules enable the reads mapping to the pseudo reference sequence which somehow overcomes the inefficiency of multiple mappings that adopt by the existing error correction methods.

**Conclusions:**

To verify the performance of our method, we selected *Canu* and *Jabba* to compare with *QIHC* in several aspects. As a hybrid correction method, we first conducted a groups of experiments under different coverages of the next-generation sequencing data. *QIHC* is far ahead of *Jabba* on accuracy. Meanwhile, we varied the coverages of the third generation sequencing data and compared performances again among Canu, Jabba and QIHC. *QIHC* outperforms the other two methods on accuracy of both correcting the sequencing errors and identifying the heterozygous sites, especially at low coverage. We carried out a comparison analysis between *Canu* and *QIHC* on the different error rates of the third generation sequencing data. *QIHC* still performs better. Therefore, *QIHC* is superior to the existing error correction methods when heterozygous sites exist.

## Background

Genomic researches have been revolutionized by the genome sequencing technology, especially the single-molecule long-read sequencing technology, also called the third-generation sequencing (TGS) [1]. The emergence of TGS technology not only inherits the high throughput of the next-generation sequencing (NGS), but also produces longer reads with the lengths greater than 10kbp compared to NGS reads which are generally limited to 100bp [1–8]. TGS has also brought a huge boost to a number of fields, such as detecting structural variations [9, 10], identifying methylations [11–13], and further facilitating disease diagnoses [14]. Although TGS is on the cutting edge in read length and many other aspects, its sequencing error rate falls behind NGS due to its technical limitations. For example, one of the key sequencing technologies of TGS is to identify the spectrum caused by different nucleotides passing a nanopore, during which it is possible to misidentify the current nucleotides as deletions or insertions when an abnormal speed occurs [15–17]. More importantly, in terms of research value, the importance of TGS has been steadily increasing, and its sequencing error rate has also been gradually decreasing. The PB-scale third-generation sequencing data, which rapidly accumulated in the past decade, cannot be discarded. It is considered that the sequencing errors can be corrected by algorithmic methods.

Along with the development of TGS, bioinformatics researchers have been gradually focusing on correcting sequencing errors by error correction algorithms. A bunch of algorithms have emerged. With continuous optimization and development, the existing error correction methods have performed well on overall accuracy, although the performance at heterozygous loci is not satisfactory [18, 19]. However, heterozygous variations are more common than homozygous variations in many cases, and heterozygosity plays a valuable role in disease genotype-phenotype analyses and genetics research. But sequencing error correction becomes more complicated in the presence of heterozygosity, the existing methods encounter some challenges in handling heterozygosity. According to the given data, and the existing methods generally fall into two categories: self correction algorithms and hybrid correction ones. The input data of self correction is a set of TGS reads, long reads (LRs) for short. Its core idea is to call a consensus between LRs, which is achieved by building multiple alignments among LRs and computing local alignments [20]. It is practical to estimate heterozygous variations based on multiple alignments and local alignments, however, the coverage of LRs limits the correction performance. Currently, the coverages of the published data sets are generally low due to the cost, which results in short splicing sequences and unsatisfactory correction performance. Therefore, the low coverage of LRs limits applications of self correction [18], which also makes it more difficult to properly handle heterozygous sites. For example, when the coverage of LRs is lower than 2, considering from the perspective of mathematical expectation, it is impossible to distinguish a heterozygous variation from sequencing errors, even from homozygous variations.

**Fig. 1:**
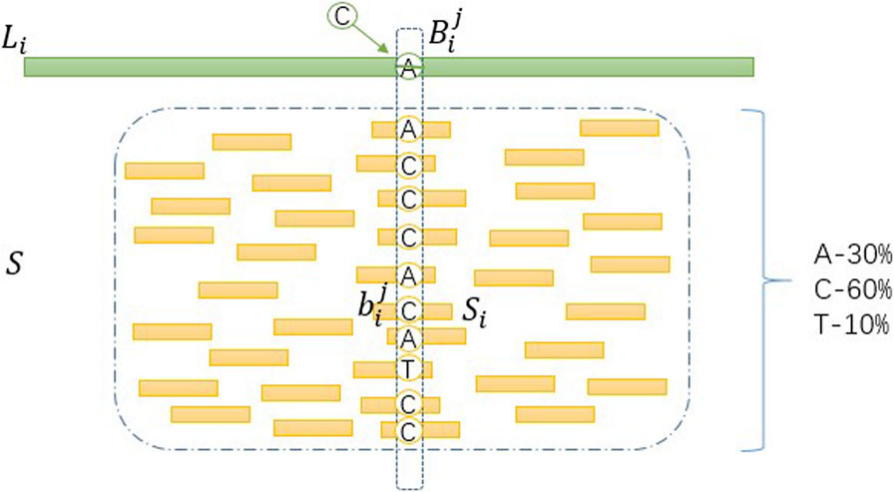
The voting rule in existing hybrid correction methods incorrectly handle heterozygous

Because of the problems of self correction, hybrid correction is more popular in practice. The basic idea of hybrid correction is: given LRs and a set of NGS reads, for simplicity, called short reads (SRs), map SRs to a read that extracted from LRs, then vote with the mapping results of SRs, the allele with the most votes is the final correction result [21]. It can be seen that the core of this basic idea is voting, and some latest researches have also improved the voting process. According to this idea, the reason why the current hybrid correction algorithms cannot solve the heterozygous condition lies in the structure of the algorithms themselves. In the case of hybrid correction represented by *proovread* [22] and *ECTools* [23], the heterozygous variations are not considered as special situations in voting process. Figure 1 shows an example of miscorrection. Furthermore, even if the heterozygous variations are considered on SRs, since each long read (LR) is treated independently, the purpose of distinguishing the heterozygous variations and the noise cannot be achieved. On the other hand, the objective of the algorithms is to correct LRs, so the coverage of SRs is low in order to control the cost, which is also not conducive to the identification of heterozygosity. In addition, all SRs need to be mapped again for each LR, which leads to low correction efficiency. All these make the existing hybrid correction methods at a disadvantage when dealing with heterozygous sites.

Distinguishing a heterozygous site from sequencing errors is the key and difficult point for properly dealing with heterozygosity, which makes the simple voting process impossible to handle the complicated condition. Taking into account the characteristics of the heterozygous variation and the limitations of the existing correction methods, we propose a novel hybrid correction method, named *QIHC*. The highlight of *QIHC* distinguishing it from the existing methods is the adoption of probabilistic models, which solves the problem that the existing methods cannot effectively deal with error correction of heterozygous genomic LRs to a great extent. Specifically, according to the sequencing principle of reads, we can assume that bases of reads mapped to the same site obey binomial distribution. Since the bases for mapping are respectively derived from LRs and SRs, the probability in the binomial distribution is related to the sequencing error rate, in general, the prior error rate of LRs ranges from 15% to 20%, and the prior error rate of SRs is around 5% [15–17, 24]. Therefore, we propose two sets of probabilistic models based on Bayesian classifer for LRs and SRs respectively, which differ from the different sequencing error rates and judge heterozygosity of the mapping sites by calculating the posterior probabilities before voting. More specifically, a set of probabilistic models determines whether a position is homozygous or heterozygous by obtaining the maximum posterior probability. Then, according to the heterozygosity judgment, the corresponding site is corrected by a voting mechanism. Compared to the existing methods, *QIHC* has better correction performance when using the probabilistic models to judge heterozygosity before voting than simply voting. Similarly, another set of probabilistic models works on self correction module, which makes the results obtained under low coverage more excellent than directly voting.

Through application of the above two sets of probabilistic models, the correction of LRs is realized, and a completed data process flow is formed. In this paper, we compared *QIHC* with two methods called *Canu* [25] and *Jabba* [26] and designed five groups of experiments, which respectively compared the influence of coverage of SRs on accuracy, the influence of error rate of LRs on accuracy, the accuracy and the heterozygosity quality of the different correction methods, and the potential effects of the different prior probability distributions on the performance. Taking a set of accuracy comparison experiments, when the coverage is 3 ×, the accuracy spans from 10.2% of *Jabba* to 72.4% of *Canu*, and finally to 87.8% of *QIHC*. From the experiment results, our method can always achieve excellent results at low coverage, whether it is LRs or SRs coverage.

## Results

### Experimental protocol

Let ***L*** denote a set of TGS reads and ***S*** denote a set of NGS data, respectively. To demonstrate performance of *QIHC* at heterozygous positions, we performed experiments on several aspects. Overall,

1. We performed our experiments on the following datasets: the third-generation sequencing data ***L*** with coverage of 3 ×, 5 ×, 10 ×, 12 × and 15 ×, respectively; the next-generation sequencing data ***S*** with coverage of 5 ×, 10 ×, 15 ×, 20 × and 50 ×, respectively. It should be noted that all third-generation sequencing datasets used in our experiments contain 500 heterozygous variations. For a position with heterozygous variation, we say that this position has heterozygosity. We generated these data under different configurations by *PBSIM* [27], specifically, a portion of the human genome *hg19* was taken as a reference genome for generating simulation data, we called the reference genome *hg19_ref*. In view of *BLASR*’s fault tolerance and strong alignment ability [28], we chose *BLASR* as the alignment tool. The parameters we set for *BLASR* were: *-header*, *-m* 5.
2. Except for *Canu*, we did not make too many comparisons with other error correction methods such as *FMLRC* [29], *LoRDEC* [20] or *HALC* [30], because the experiments at heterozygous positions had already done in the literature [18], and the performance of these methods on correcting bases at heterozygous positions was proven not to be ideal. The reason for choosing *Canu* was that from 2015 to the present, the version of *Canu* was from 1.0 to 1.8, and continuous improvement had made *Canu* a stable and widely-used error correction tool. It is worth mentioning that *Canu* v1.8 added a module called trio bunning that specializes in handing heterozygous conditions. Therefore, it would be more convincing for us to choose *Canu* to compare.

### Evaluation strategies

In order to show the error correction results efficiently and pertinently, we only demonstrated the results of the sites with heterozygous variation here. For each heterozygous position, we investigated the change of its heterozygosity after error correction. Specifically, the criteria for judging whether the site is still heterozygous is as follows: mapping the corrected long reads set to *hg19_ref*, observing the distribution of corresponding bases mapped to the heterozygous position, if the distribution satisfies heterozygosity, then the position remains heterozygosity; otherwise, its heterozygosity is lost. True positive (TP) positions are those heterozygous sites that maintain heterozygosity after correction, whereas false negative (FN) positions are the sites with original heterozygosity that cannot remain heterozygosity after correction, whether it is noise or homozygous. To evaluate the error correction performance of different error correction methods and different coverages in the TGS data with heterozygous variations, we focused on accuracy, which was computed by 1-*errorrate*.

### Analysis of experimental results

#### Analysis of accuracy under different coverages of NGS data

**Fig. 2:**
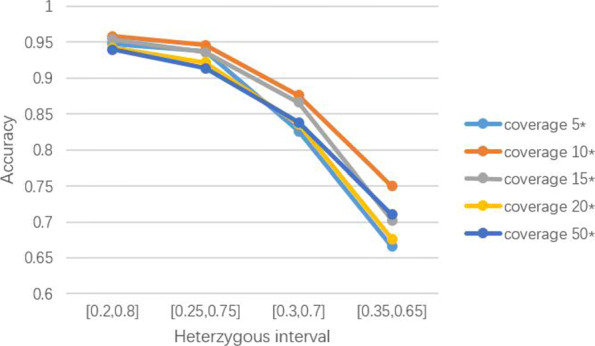
Accuracy of QIHC with different coverages of S

*QIHC* requires the participation of ***S***, so it is necessary to confirm the impact of different coverages of ***S*** on the correction results. ***S*** was generated from *hg19_ref* when the coverages were 5 ×, 10 ×, 15 ×, 20 × and 50 × by *PBSIM*, respectively. ***L*** was also derived from *hg19_ref*, its coverage was set to 5 × in consideration of runtime. Figure 2 shows the accuracy values of *QIHC* influenced by the coverage of ***S***.

In order to analyze the results shown in Fig. 2, “heterozygous interval” needs to be described first. The heterozygous interval defines what conditions need to be satisfied if the base distribution mapped to the heterozygous variation site is identified as retaining heterozygosity. For example, when the heterozygosity interval is set to [0.2,0.8], a heterozygous variation site is considered to retain heterozygosity only when the bases distribution under this site falls within the interval. The interval [0.2,0.8] is a heterozygous interval generally recognized in the field of bioinformatics [31]. Here, for the sake of more illustrative experiments, we also selected [0.25,0.75], [0.3,0.7] and [0.35,0.65] as other intervals. Since *Canu* does not need to input ***S***, we chose *Jabba* to compare with *QIHC* under different coverages of ***S***, Table 1 shows the results. As can be seen from the results, *QIHC* performed much better than *Jabba*, specifically, the difference value of accuracy was up to 85.6% when coverage of ***L*** was 5 × and heterozygous interval was [0.2,0.8]. According to Fig. 2, *QIHC*’s correction accuracy reached best when the coverage was 10 ×. It can be seen that *QIHC* is not sensitive to the coverage of ***S***, which facilitates the use of lower coverage ***S*** for the purpose of correction ***L***.

**Table 1:** The comparisons on accuracy between ***QIHC*** and ***Jabba***

		Coverage of ***S***				
Heterozygous interval		5 ×	10 ×	15 ×	20 ×	50 ×
[0.20,0.80]	*QIHC*	0.948	0.958	0.954	0.942	0.940
	*Jabba*	0.092	0.230	0.322	0.316	0.288
[0.25,0.75]	*QIHC*	0.938	0.946	0.936	0.922	0.914
	*Jabba*	0.086	0.206	0.290	0.294	0.258
[0.30,0.70]	*QIHC*	0.826	0.876	0.866	0.836	0.838
	*Jabba*	0.066	0.168	0.224	0.236	0.200
[0.35,0.65]	*QIHC*	0.666	0.750	0.702	0.676	0.710
	*Jabba*	0.022	0.110	0.138	0.166	0.138

#### Comparison to the existing methods on accuracy

**Fig. 3:**
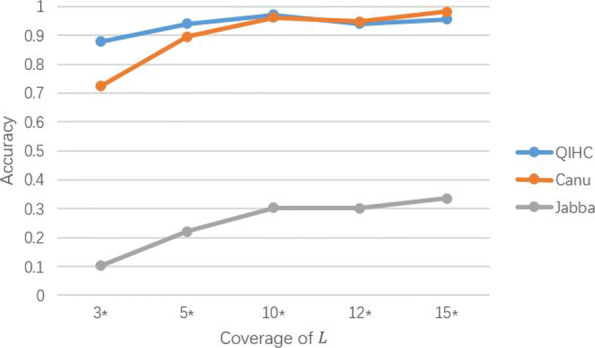
Accuracy of QIHC, Canu and Jabba

In this part of the experiment, we chose *Canu* and *Jabba* as the comparison methods. The results are shown in Table 2. It can be seen that we experimented with low coverages. The reason is that TGS technology generates a large amount of low-coverage sequencing data due to its cost, it is more practical to experiment with low-coverage data. Among the results, *Jabba* was significantly less accurate than *QIHC* and *Canu*, which also confirmed that the early error methods did not consider the heterozygous variation sites at all. For the results of *Canu* and *QIHC*, when the coverage was set to 3 ×, the accuracy of *QIHC* was up to 15 percentage points ahead of *Canu*. As the coverage increased, *QIHC*’s performance had been more excellent until the coverage reached 12 ×. After that, although *Canu* had a slight overshoot, the gap was not significant. Figure 3 graphically shows the experiment result when the heterozygosity interval was [0.25,0.75].

**Table 2:** The comparisons on accuracy among ***QIHC***, ***Canu*** and ***Jabba***

Heterozygous interval	[0.20,0.80]					[0.25,0.75]				
Coverage of ***L***	3 ×	5 ×	10 ×	12 ×	15 ×	3 ×	5 ×	10 ×	12 ×	15 ×
*QIHC*	0.888	0.954	0.978	0.95	0.972	0.878	0.940	0.972	0.940	0.956
*Canu*	0.780	0.922	0.968	0.958	0.988	0.724	0.896	0.962	0.948	0.982
*Jabba*	0.112	0.234	0.336	0.344	0.368	0.102	0.222	0.304	0.302	0.336

#### Comparison between Canu and QIHC on heterozygosity quality

In this part of the experiment, the quality of heterozygosity maintained by *Canu* and *QIHC* would be analyzed. The so-called heterozygosity quality analysis is that the corrected heterozygous site examines alleles mapped to the site after ensuring that the base distribution falls within the heterozygous interval. For example, a heterozygous site consisting of allele A and C, after correction, the bases mapped to this site should still be dominated by base A and C; otherwise, although the site remains heterozygosity within the heterozygous interval, its heterozygosity quality is very low. To more clearly analyze the heterozygosity quality, we quantify it. Specifically, for a A-C heterozygous site, we compare the proportion of base A and C mapped to this site with the proportion of base T and G. If the former is larger than the latter, that is, the difference value is positive, the heterozygosity quality of the site is high, otherwise, the quality is poor. Other types of heterozygous site are similar. Thereafter, a more detailed analysis of the sites with high heterozygosity quality is conducted to classify them as good and excellent. Specifically, the case where the difference value is between 0 and 0.3 is defined as good, and between 0.3 and 1 is defined as excellent. No doubt, excellence is better than good.

The experiment results with coverage 15 × were selected for heterozygosity quality analysis. The results are shown in Table 3. After removing the sites that did not maintain heterozygosity, 494 and 486 heterozygous sites left in *Canu* and *QIHC* results, respectively. Among the sites that maintained heterozygosity, the difference value of 246 heterozygous sites in *Canu* result were negative, that is, their heterozygosity qualities were poor; in comparison, although the number of heterozygous sites maintained in *QIHC* result was slightly less than that of *Canu*, the number of heterozygosity sites with poor quality was significantly less than *Canu*, which was 210. Similarly, *QIHC* was also significantly better than *Canu* in terms of the number of high quality heterozygous sites, 245 and 211, respectively. Among the high quality heterozygous sites, *QIHC* had a higher proportion of excellence. Therefore, from the above analysis, *QIHC* was slightly inferior to *Canu* in accuracy when the coverage was 15 ×, but after in-depth analysis, it can be concluded that *QIHC* was significantly better than *Canu* in heterozygosity quality. This is also the reason why we chose the 15 × coverage for deep analysis, that is, *QIHC* can still lead significantly in other aspects when its accuracy result is not dominant.

**Table 3:** The comparisons on heterozygosity quality between ***QIHC*** and ***Canu***

Difference value	Negative	Draw	Positive	
*QIHC*	210	31	245	
			excellence	good
			57	181
*Canu*	246	37	211	
			excellence	good
			20	191

#### Analysis of accuracy with different sequencing error rates of TGS data

In this part of experiment, we tested the accuracy of *QIHC* and *Canu* at different sequencing error rates of ***L***, the experimental results are shown in Table 4. Since *Jabba*’s correction results at heterozygous sites were much behind *QIHC* and *Canu*, *Jabba* was not used as a comparison method here. We performed comparison experiments with 20%, 15%, 10% and 5% sequencing error rates in the heterozygous intervals [0.2,0.8], [0.25,0.75], [0.3,0.7] and [0.35,0.65]. Combined with the results of *QIHC* and *Canu*, the accuracy trends in the four heterozygous intervals were roughly the same, that is, accuracy was optimal when the sequencing error rate was 20%, then accuracy declined as sequencing error rate decreased, finally, there was a rebound in accuracy when the sequencing error rate was 5%. Comparing the results of *QIHC* and *Canu*, accuracies of *QIHC* were almost all higher than *Canu*, no matter what interval and sequencing error rate.

**Table 4:** The comparisons on accuracy with different sequencing error rates of ***L*** between ***QIHC*** and ***Canu***

Heterozygous interval	[0.20,0.80]				[0.25,0.75]			
Error rate of ***L***	20%	15%	10%	5%	20%	15%	10%	5%
*QIHC*	0.920	0.868	0.882	0.882	0.902	0.850	0.862	0.870
*Canu*	0.868	0.852	0.864	0.866	0.846	0.796	0.802	0.836
Heterozygous interval	[0.30,0.70]				[0.35,0.65]			
Error rate of ***L***	20%	15%	10%	5%	20%	15%	10%	5%
*QIHC*	0.816	0.778	0.760	0.788	0.618	0.560	0.536	0.592
*Canu*	0.780	0.710	0.690	0.768	0.592	0.520	0.504	0.626

#### Analysis of potential effects of the different prior probability distributions on the performance

So far, we focused on the presentation of the overall framework of the algorithm, directly defined the homozygous and heterozygous prior probabilities as point probabilities. In this part of experiment, we further discussed the potential effects of other prior probability distributions on performance. Here we chose Beta distribution for discussion, the reasons are as follows: Beta distribution can be understood as a probability distribution of probabilities, that is, it represents all the possible values of a probability when we don’t know what that probability is. Going back to the background of our method, the prior probability *P*(*c*) is available in most cases, but in a few cases we can’t explicitly obtain *P*(*c*), which happens to be the area where Beta distribution is good at processing. Moreover, by adjusting the shape parameters in Beta distribution, the probability distribution can be made into various shapes we want, so Beta distribution is sufficient to express our estimation of the prior probabilities in advance. We made *P*(*c*) obey Beta distribution, according to the principle of Beta distribution, $P(c)=\frac {{\theta }^{a-1}{(1-\theta)}^{b-1}}{B(a,b)}$, where *a* and *b* are shape parameters, *θ* is a reasonable guess of the probability of homozygosity or heterozygosity derived from experiences in previous studies, Beta function $B(a,b)=\frac {\Gamma (a)\Gamma (b)}{\Gamma (a+b)}$, where gamma function is defined as $\Gamma (x)={\int \nolimits }_{0}^{\infty }t^{x-1}e^{-t}dt$.

**Fig. 4:**
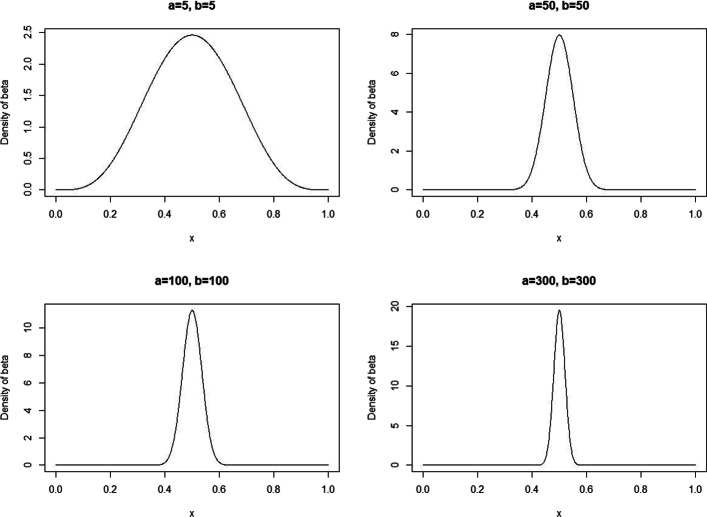
Density of four different Beta distributions

Based on the characteristics of Beta distribution, we varied the probability density distribution by changing values of the shape parameters *a* and *b*, and observed the potential effects of the different prior probability distributions on the performance. Specifically, we made the expected value of the distribution equal to 0.5 (that is, $\frac {a}{a+b}=0.5$), which means the probability of homozygosity will most likely around 0.5, but it could reasonably carry out small fluctuations. Users can set this value according to their actual situations. Here, Fig. 4 shows four Beta distributions with the expected value equal to 0.5 by changing *a* and *b* values. We can see that as the values of *a* and *b* increase, the curve becomes more “sharp”, that is, the probability distribution of the prior probability is more concentrated around the expected value. We performed comparisons experiments with 0.1, 0.25, 0.35, 0.5 and 0.75 prior probabilities of homozygosity in the heterozygous intervals [0.20,0.80], [0.25,0.75] and [0.30,0.70]. Since only *QIHC* involves the prior probability distribution, we just did comparative experiments on it, the experimental results are shown in Table 5. Through the results we can see, when the prior probability was from 0.25 to 0.75, which could be the common practice, the accuracy decreased slightly as the prior probability was far from the expected value, but it still maintained a relatively stable state. Specifically, in the heterozygous interval [0.2,0.8], the accuracy decreased from 0.964 to 0.95, then to 0.94, and the corresponding prior probabilities were 0.5, 0.35, and 0.25, respectively. Similarly, when the prior probability changed in the opposite direction to 0.75, the accuracy also reduced to 0.952. Further, when the prior probability continued to drop to 0.1, the accuracy fell to 0.652, which means the accuracy of the proposed method may be attenuated when the prior probability is at extrem level. Through the testimony of the experimental results, we can conclude that the accuracy reaches the optimum at the expected value of Beta distribution, then, as the prior probability get further away from the expected value, the accuracy gradually deteriorate. Obviously, the smaller the fluctuation of the prior probability, the smaller the impact on the posterior probability calculation, that is, the more stable the performance of *QIHC*. For example, in the case of *a*=*b*=5, the prior probability is even possible to be more extreme, such as 0.1, although this situation is less likely, it also increases the instability of the correction result. When shape and scale parameters are quite large (e.g. *a*=*b*=300), the prior probability reasonably ranges from 0.45 to 0.55, which has little effect on the performance.

**Table 5:** The comparisons on accuracy with different prior probabilities of homozygosity on ***QIHC***

Heterozygous interval	[0.20,0.80]	[0.25,0.75]	[0.30,0.70]
Prior probability			
0.10	0.652	0.638	0.576
0.25	0.940	0.936	0.844
0.35	0.950	0.936	0.872
0.50	0.964	0.944	0.866
0.75	0.952	0.940	0.868

## Conclusions

The third-generation sequencing (TGS) technology has demonstrated unique advantages in terms of read length and so on, which providing great convenience for downstream analysis. As we start to see the promising potential of TGS, we must also be aware of where it might stumble. High sequencing error rate is a major problem in TGS technology, therefore, correcting the sequencing errors is an inevitable step when we apply the TGS data. The existing error correction methods are quite complete for the correction strategy at normal sites, but they are often not considered in the correction of heterozygous variation positions, which is an aspect that cannot be ignored. We have therefore proposed a method to break this limitation, solving the error correction at heterozygous sites. Our novel error correction method, termed *QIHC*, adopts probabilistic models to deal with heterozygous variantion sites based on the advantages of the existing error correction methods. According to the sequencing principle, *QIHC* reasonably assumes that the mapping bases obey binomial distribution, uses Bayesian classifier to judge the heterozygosity of sites by calculating the posterior probabilities, and then performs error correction. In addition, *QIHC* also generates a pseudo reference sequence, which makes our algorithm suitable for genomic data without reference sequences, and achieves high efficiency of single mapping and repeated using. In the simulation experiments, *QIHC* performs significantly better than *Canu* and *Jabba* at heterozygous variantion sites, especially in the case of low coverage. From the comparison of *Canu* and *QIHC*, the performance of *QIHC* at low coverage is significantly superior to that of *Canu* in all aspects; as the coverage increases to 15 ×, the accuracy of *QIHC* is also greatly improved, although *Canu* has the slightly upper hand in accuracy after eliminating the interference of low coverage, but still far behind *QIHC* in terms of the heterozygosity quality. In the case of low coverage, since *Canu-correct* is a self correction algorithm, coverage of the TGS data is a key factor affecting performance of *Canu*, making its performance worse than *QIHC* in many aspects; with the coverage going up, *Canu* continues the principle of *Celera Assembler* and adopts “Overlap-Layout-Consensus”, that is, after the overlap of sequences, voting correction is performed directly according to the “the minority is subordinate to the majority” rule. *QIHC* adds probabilistic models for judging heterozygosity, so that even when the accuracy is slightly backward, it can prevail in the heterozygosity quality. For future work, we will try several assembly tools and generate the contigs to optimize the correction results of *QIHC* as much as possible. Moreover, we will adjust the program code to optimize running time and memory consumption.

## Methods

Let ***L*** denote a set of TGS reads and ***S*** denote a set of NGS data, respectively. Suppose that we are given ***L*** and ***S***, *QIHC* uses the probabilistic models to judge the heterozygosity through Bayesian classifier, and corrects reads from ***L*** based on the integration of self correction and hybrid correction mechanism, finally outputs the corrected set ***L’***. No reference sequence is required for the inputs. From the inputs to the outputs, *QIHC* includes three major modules, which are in turn:

1. Generating pseudo reference sequence. Specifically, a pseudo reference sequence is obtained through assembly process, which can be done by any popular long-read assembly tool. Through this module, the inefficiency of repeatedly mapping ***S*** to each read from ***L*** in hybrid correction is solved. At the same time, there is no need to narrow down the *QIHC* scope of application in order to input a native reference sequence.
2. Obtaining read alignment. Simultaneously mapping ***S*** and ***L*** to the pseudo reference sequence, named *Ref*. Let ***Lm*** denote a set of TGS reads which successfully map to *Ref*, and ***Lu*** denote a set of TGS reads which do not map to *Ref*. Specifically, mapping ***L*** to *Ref* to get ***Lm*** and ***Lu***. A standard was set to accomplish this task. Dividing ***L*** into these two parts facilitates subsequent implementation of targeted correction strategies. For another, *Ref* provides anchor points for the mapping of ***Lm*** and ***S***, that is, the sites on *Ref* sever as anchor points to ensure that the corresponding bases of ***Lm*** and ***S*** are mapped to the same site.
3. Judging heterozygosity and correcting reads. This module consists of judging heterozygosity by the probabilistic models and performing the different correction strategies according to the judgment results. Specifically, after the first two modules are completed, the mutual relationships and the mapping positions between reads can be calculated. At this time, the probabilistic models can be used to calculate the posterior probabilities, furthermore, Bayesian classifier is used to judge heterozygosity, that is, the largest posterior probability is selected for decision making, finally the targeted correction strategies are implemented. This is our core module, under the premise of not losing accuracy and greatly increasing the sensitivity to heterozygosity, *QIHC* accomplishes the error correction of ***L***.

### Generating pseudo reference sequence

As the beginning of the method, we perform sequence assembly to get a pseudo reference sequence, the assembly process is as following four steps:

- : Load reads from ***L*** and align all reads to each other to get a directed graph, where each read is treated as a node.
- : Compute overlaps between any two reads based on Smith-Waterman algorithm and obtain the information of all possible overlaps. Specifically, we set the user parameters *min_length*, *max_length* and *θ* as the minimum length, the maximum length and a threshold score of overlap, respectively. Using the Smith-Waterman algorithm to compute the score of overlap between any pair of reads. Of course, if there is no overlap between two reads, the corresponding score is 0, and the overlap length is also 0. When the overlap length of a pair reads is between *min_length* and *max_length*, and the score is greater than *θ*, the overlap is established.
- : According to the overlaps, the reads from ***L*** are preliminarily assembled, and get the combined relationship of fragments, defined as contig.
- : Scan again, if there are overlaps in contigs, merge the contigs to form a new contig, and delete the original contigs. In this way, we get the final contigs.

Finally, we link these contigs to obtain a pseudo reference sequence–*Ref*. Since assembly principle of *Canu* can achieve the purpose of our assembly idea, and *Canu* adds correction and trimming before assembly to get high quality contigs, we choose *Canu* as the assembly tool.

### Obtaining read alignment

After obtaining *Ref*, ***L*** and ***S*** are mapped to the pseudo reference sequence by *BLASR* [28], which has a strong fault tolerance and can map almost all reads to *Ref*. Since *BLASR* may generate multiple mapping results for a read and sort by the percentage of mapped base, we reserve the best mapping result for each read and divide ***L*** into ***Lm*** and ***Lu*** according to the percentage of mapped base, that is, the critical value of the percentage is 90%, a read with the percentage over 90% is assigned to ***Lm***, otherwise it is assigned to ***Lu***. Next, it is necessary to map ***S*** to each read of ***Lu*** separately in order to perform a correction strategy different from ***Lm***.

### Judging heterozygosity and correcting reads

The highlight of *QIHC* is the heterozygosity judgment, the core idea is as follows: two sets of the probabilistic models are established for ***S*** and ***L*** respectively, the probabilistic models are proposed based on Bayesian classifier. According to the basic principle of Bayesian classifier, we calculate the posterior probabilities of homozygosity and heterozygosity respectively, and take the side with higher probability value as the judgment result. More specifically, the comparison of the posterior probabilities is equivalent to the comparison of the product of the prior probability and the conditional probability of heterozygosity. The prior probability is a fixed value obtained through sequencing data features, the conditional probability is subject to binomial distribution. Alleles are sorted according to the frequency of occurrence. For heterozygous cases, the first two rank are taken as the heterozygous alleles, and the rest are sequencing errors; for homozygous cases, the first rank is taken as the homozygous allele, similarly, the rest are sequencing errors. Each allele obeys its respective binomial distribution, and the latter term is calculated on the basis of the former term.

**Fig. 5:**
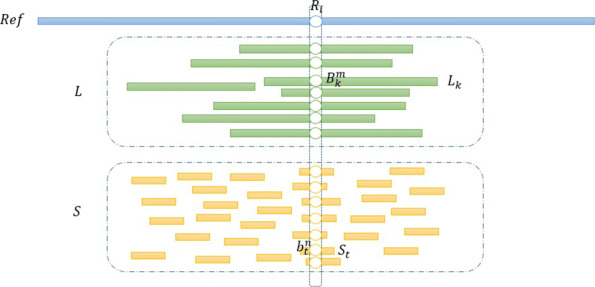
The dependency relationships and distribution among *Ref*, $ L_{k}, S_{t},B_{k}^{m}, b_{t}^{n}$ and *R*_*i*_

Specifically, the heterozygosity judgment process is described in detail with respect to the site *i* on *Ref*. Let $L_{k}, S_{t}, B_{k}^{m}, b_{t}^{n}, R_{i}$ represent the *k*th LRs, the *t*th SRs, the *m*th base of *L*_*k*_, the *n*th base of *S*_*t*_, the *i*th base of reference sequence *Ref*, respectively. Figure 5 intuitively shows the dependency relationships and the distribution among *Ref*, $ L_{k}, S_{t}, B_{k}^{m}, b_{t}^{n} $ and *R*_*i*_. According to the known knowledge, a base may be four single-bases or null. Thus, $ B_{k}^{m} $ and $ b_{t}^{n} $ have five possible alleles, which are A, T, G, C and null label N. The mapping result is subdivided, the number of long reads mapped to *R*_*i*_ is defined as the read depth of long reads, represented by *RD*_*i*_. Similarly, the read depth of short reads is defined, denoted by *rd*_*i*_. Processing the long reads which mapped to *R*_*i*_, let *X*_*q*_ denote alleles which are sorted according to the frequency of occurrence from large to small, |*X*_*q*_| denote the corresponding frequency, *q*=1, 2, 3, 4, 5. Then, we can draw the binomial distribution for *X*_*q*_: *X*_*q*_∼*Bin*(*D*_*q*_,*P*_1_), where $f\left (X_{1}|P_{1}\right)={C}_{D_{q}}^{|X_{q}|} \times {\left (P_{1}\right)}^{|X_{q}|} \times {\left (1-P_{1}\right)}^{{D_{q}}-{|X_{q}|}}, D_{q}=\sum _{q}^{5} |X_{q}|$. Similarly, the alleles and frequency of occurrence of the alleles are defined in short reads, denoted as *x*_*q*_ and |*x*_*q*_|, *q*=1, 2, 3, 4, 5. Drawing the binomial distribution for *x*_*q*_: *x*_*q*_∼*Bin*(*d*_*q*_,*P*_2_), where $f\left (X_{1}|P_{2}\right)={C}_{d_{q}}^{|x_{q}|} \times {\left (P_{2}\right)}^{|x_{q}|} \times {\left (1-P_{2}\right)}^{{d_{q}}-{|x_{q}|}}, d_{q}={\sum \nolimits }_{q}^{5} \left |x_{q}\right |$. It should be noted here that *P*_1_ and *P*_2_ are the prior probability values of *X* and *x* respectively, which vary according to the different situations, and the details are given in the probability model calculation part.

Therefore, the posterior probability of ***L*** can be calculated by Eq. (1),

1 $$\begin{array}{*{20}l} & \ \ \ \ P\left(c|X_{1},X_{2},X_{3},X_{4},X_{5}\right) \notag \\ &=P\left(X_{1},X_{2},X_{3},X_{4},X_{5}|c\right) \times P(c) \notag \\ &=P\left(X_{1}|c\right)\times P\left(X_{2}|X_{1},c\right)\times P\left(X_{3}|X_{1},X_{2},c\right)\\&\times P\left(X_{4}|X_{1},X_{2},X_{3},c\right)\times P\left(X_{5}|X_{1},X_{2},X_{3},X_{4},c\right)\times P(c) \end{array} $$

where the value of *c* is homozygosity or heterozygosity. The probability of ***S*** is similar. For the allele whose occurrence frequency is 0, the corresponding item is removed in actual calculation. So far, two kinds of the posterior probabilities are obtained by multiplying the above probabilities, which are the probabilities of observing the bases distribution when homozygosity and heterozygosity; inferring the heterozygosity of the site based on the maximum probability value.

**Fig. 6:**
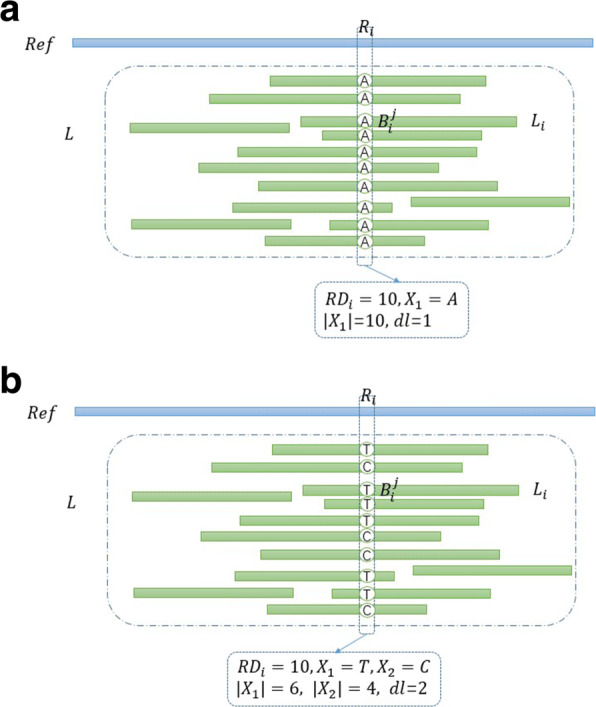
The possible distributions of bases

Then, the bases distribution as a new definition is brought up, which reflects how many kinds of alleles are mapped to *R*_*i*_, denoted as *dl*, which can be computed as

2 $$\begin{array}{*{20}l} dl=\sum\limits_{n=1}^{5} I \left(|X_{q}| \ne0 \right) \end{array} $$

where *I*(·) is an indicator function, which outputs 1 when the equation is true. Similarly, the bases distribution are defined in short reads, denoted as *ds*. The possible distributions of bases are given in Fig. 6, which contribute to understanding of the heterozygosity judgment.

After |*X*_*q*_|,|*x*_*q*_|, *dl*, *ds*, *RD*_*i*_ and *rd*_*i*_ are calculated, the heterozygosity judgment is performed. Let *D*_*i*_ represent the bases distribution under the site *i* we observed. According to the bases distribution, it can be divided into five cases: *d*=1, *d*=2, *d*=3, *d*=4 and *d*=5, *d* here refers to *dl* and *ds*.

Case 1:

If *dl*=1, then it is directly judged to be homozygosity.

If *ds*=1, then it is directly judged to be homozygosity.

Case 2:

If *dl*=2, thus |*X*_1_|+|*X*_2_|=*RD*_*i*_, then the posterior probability of *D*_*i*_ under homozygosity and heterozygosity are as follow (see the corresponding details of calculation formulas in Additional file 1):

3 $$\begin{array}{*{20}l} P \left(i\ is\ homozygosity|D_{i}=\left\{X_{1},X_{2}\right\} \right) \end{array} $$

4 $$\begin{array}{*{20}l} P\left(i\ is\ heterozygosity|D_{i}=\{X_{1},X_{2}\}\right) \end{array} $$

After calculating the two posterior probabilities, the result of judgment is corresponding to the larger value.

The judgment principle of ***S*** is similar to ***L***, we do not describe here, see the corresponding details in Additional file 1.

Case 3:

If *dl*=3, thus |*X*_1_|+|*X*_2_|+|*X*_3_|=*RD*_*i*_, then the posterior probabilities of homozygosity and heterozygosity are given below (see the corresponding details of calculation formulas in Additional file 1):

5 $$\begin{array}{*{20}l} P\left(i\ is\ homozygosity|D_{i}=\{X_{1},X_{2},X_{3}\} \right) \end{array} $$

6 $$\begin{array}{*{20}l} P\left(i\ is\ heterozygosity|D_{i}=\{X_{1},X_{2},X_{3}\}\right) \end{array} $$

Similar to the case with *d*=2, we take the larger value in Eqs. (5) and (6) as the judgment result of ***L***.

Case 4:

If *dl*=4, thus |*X*_1_|+|*X*_2_|+|*X*_3_|+|*X*_4_|=*RD*_*i*_, then the posterior probabilities of homozygosity and heterozygosity are given below (see the corresponding details of calculation formulas in Additional file 1):

7 $$\begin{array}{*{20}l} P\left(i\ is\ homozygosity|D_{i}=\{X_{1},X_{2},X_{3},X_{4}\}\right) \end{array} $$

8 $$\begin{array}{*{20}l} P\left(i\ is\ heterozygosity|D_{i}=\{X_{1},X_{2},X_{3},X_{4}\}\right) \end{array} $$

Case 5:

If *dl*=5, thus |*X*_1_|+|*X*_2_|+|*X*_3_|+|*X*_4_|+|*X*_5_|=*RD*_*i*_, then the posterior probabilities of homozygosity and heterozygosity are given below (see the corresponding details of calculation formulas in Additional file 1):

9 $$\begin{array}{*{20}l} & \ \ \ \ P\left(i\ is\ homozygosity|D_{i}=\{X_{1},X_{2},X_{3},X_{4},X_{5}\}\right) \notag \\ & =P\left(D_{i}=\{X_{1},X_{2},X_{3},X_{4},X_{5}\}|i\ is\ homozygosity\right)\\&\quad\times P\left(i\ is\ homozygosity\right) \notag \\ & = f\left(X_{1}|P_{1}\right) \times f\left(X_{2}|P_{2}\right) \times f\left(X_{3}|P_{3}\right) \times f\left(X_{4}|P_{4}\right) \\&\quad\times f\left(X_{5}|P_{5}\right) \times P\left(i\ is\ homozygosity\right) \end{array} $$

10 $$\begin{array}{*{20}l} & \ \ \ \ P\left(i\ is\ heterozygosity|D_{i}=\{X_{1},X_{2},X_{3},X_{4},X_{5}\}\right) \notag \\ & =P\left(D_{i}=\{X_{1},X_{2},X_{3},X_{4},X_{5}\}|i\ is\ heterozygosity\right)\\&\quad\times P\left(i\ is\ heterozygosity\right) \notag \\ & = f\left(X_{1}|P_{1}\right) \times f\left(X_{2}|P_{2}\right) \times f\left(X_{3}|P_{3}\right) \times f\left(X_{4}|P_{4}\right) \\&\quad\times f\left(X_{5}|P_{5}\right) \times P\left(i\ is\ heterozygosity\right) \end{array} $$

So far, the strategy of the heterozygosity judgment has been given. In general, the input to this process is the bases distribution under site *i*, and the different probabilistic models are implemented for different sources of reads. The final output is the result of heterozygosity of site *i*.

Then, we perform the different correction strategies for ***Lm*** and ***Lu***, respectively.

For the correction of ***Lm***, the inputs are the bases distributions of ***Lm*** and ***S*** and their heterozygosity judgment results. Correcting ***Lm*** is our goal, so the *R*_*i*_ on *Ref* is only used as an anchor point to locate related reads of ***Lm*** and ***S***, as shown in Fig. 5. Under the same anchor point *R*_*i*_, results of heterozygosity judgment for the bases distributions produce four possible combinations: heterozygous result from ***Lm*** and homozygous result from ***S***; heterozygous result from ***Lm*** and heterozygous result from ***S***; homozygous result from ***Lm*** and homozygous result from ***S***; homozygous result from ***Lm*** and heterozygous result from ***S***. For these four combinations, *QIHC* makes a decision: when the judgment results of ***Lm*** and ***S*** are consistent, since the sequencing accuracy of NGS is much higher than that of TGS, the judgment result of ***S*** is adopted; otherwise, the party whose judgment result is homozygosity is accepted. Thus, the final judgment result of heterozygosity is obtained, which is defined as *H*_*m*_. According to *H*_*m*_, the following correction rules are implemented:

If *H*_*m*_ is homozygosity, then the site to be corrected is replaced with the allele which appears most frequently among bases mapped to *R*_*i*_;

If *H*_*m*_ is heterozygosity: if the site to be corrected is already one of the top two frequent alleles among bases mapped to *R*_*i*_, then leave the allele of this site as it is; otherwise, the site to be corrected is randomly replace with one of the top two frequent bases.

According to the above decision results, all reads corresponding to the anchor point in ***Lm*** are corrected by the correction rules, a correction result set ***Lm’*** is outputted.

For the correction of ***Lu***, since ***Lu*** is the set of long reads that have not been successfully aligned to *Ref*, it can be seen that there is not enough correlation between each read in ***Lu***. Therefore, the inputs are the bases distributions of ***S*** mapped to reads of ***Lu*** and their heterozygosity judgment results, using ***S*** to correct each read in ***Lu*** one by one. The basic principle is obtaining the final heterozygosity judgment result of ***S*** named *H*_*u*_, and correcting ***Lu*** according to the following criterions:

If *H*_*u*_ is homozygosity, then the site to be corrected is replaced with the allele which appears most frequently among bases mapped to the base $B_{k}^{m}$;

If *H*_*u*_ is heterozygosity: if the base corresponding to the site to be corrected is already one of the top two frequent alleles among bases mapped to $B_{k}^{m}$, then leave the base of the site as it is; otherwise, the site to be corrected is randomly replaced with one of the top two frequent bases.

It is worth noting that the implementation of heterozygosity judgment and correction rules here only use the information provided by ***S***. All reads in ***Lu*** are corrected, a correction result set ***Lu’*** is outputted. Eventually, ***Lm’*** and ***Lu’*** form ***L’*** together.

Overall, we design an error correction algorithm with high sensitivity to heterozygosity, the algorithm mainly consists of the following steps:

- : Assemble ***L*** and get contigs;
- : Link contigs one by one and obtain a pseudo reference sequence–*Ref*;
- : Map ***L*** to *Ref* and get ***Lm*** and ***Lu***;
- : Map ***S*** to each read of ***Lu***, obtain *rd*_*i*_,*os*(*V*_*n*_) and *ds*, implement heterozygosity judgment and save result;
- : Map ***Lm*** to *Ref*. For *R*_*i*_ of *Ref*, obtain *RD*_*i*_,*ol*(*V*_*n*_) and *dl*, implement heterozygosity judgment and save result;
- : Map ***S*** to *Ref*. For *R*_*i*_ of *Ref*, obtain *rd*_*i*_,*os*(*V*_*n*_) and *ds*, implement heterozygosity judgment and save result;
- : Make the final judgment *H*_*m*_ for ***Lm***, if the results of step 5 and step 6 are consistent, the result of step 6 is adopted; otherwise, the step whose result is homozygosity is accepted, jump to step 9;
- : According to the result of step 4 and the correction rules mentioned above, correct each read of ***Lu***, obtain the correction set ***Lu’***;
- : According to the result of step 7 and the correction rules mentioned above, correct all reads of ***Lm*** which corresponding to the anchor point *R*_*i*_, then load *R*_*i*+1_ and jump to step 5, until all sites on *Ref* are traversed, obtain the correction set ***Lm’***;
- : Combine ***Lu’*** and ***Lm’*** to get ***L’***.

## Supplementary information

**Additional file 1** Supplemental Material 1 — The corresponding details of calculation formulas.

## Data Availability

The source codes and related data have been uploaded and maintained at https://github.com/LiuJiaqiqi/QIHCfor academic use only.
