# Supplementary material for: A hybrid correcting method considering heterozygous variations by a comprehensive probabilistic model
Source: BMC Genomics. 2020 Nov 18;21(Suppl 10):753. doi: 10.1186/s12864-020-07008-9 (PMC7677778; doi:10.1186/s12864-020-07008-9)
Supplement: Supplementary file 1 — Additional file 1 Supplemental Material 1 — The corresponding details of calculation formulas. [file 12864_2020_7008_MOESM1_ESM.pdf]

Supplemental Material

Supplemental Material 1 — The corresponding details of calculation formulas

A consensus needs to be reached: according to the sequencing error rate, the noise of $\boldsymbol{S}$ is subject to the binomial distribution of p=0.05 by default; the noise of $\boldsymbol{L}$ is subject to the binomial distribution of p=0.2, users can change p based on the actual situation of data. Let D_i represent the bases distribution under the site i we observed. According to the bases distribution, it can be divided into five cases: d=1, d=2, d=3, d=4 and d=5, d here refers to dl and ds. Giving four probabilities first, p_1, p_2, which in turn represent the sequencing error rates of TGS technology and NGS technology, in general, we make p_1=0.2, p_2=0.05; p_3, p_4, which in turn represent the prior probabilities of homozygous and heterozygous conditions. For the sake of simplicity, we represent the values of $|X_1|$ to $|X_5|$ as r_1 to r_5.

Case 1:

If dl=1, then it is directly judged to be homozygosity.

If ds=1, then it is directly judged to be homozygosity.

Case 2:

If dl=2, thus $r_1 + r_2 = RD_i$, then the posterior probability of D_i under homozygosity is as follow:

$$\begin{aligned} &P\left(i\ is\ homozygosity|D_i = \{X_1, X_2\}\right) \\ &= P\left(D_i = \{X_1, X_2\}|i\ is\ homozygosity\right) \times P\left(i\ is\ homozygosity\right) \\ &= C_{RD_i}^{r_1} \times (1-p_1)^{r_1} \times {p_1}^{RD_i - r_1} \times C_{RD_i - r_1}^{r_2} \times {p_1}^{r_2} \times (1-p_1)^{RD_i - r_1 - r_2} \times p_3 \end{aligned} \tag{1}$$

where $C_a^b = \frac{a!}{b!(a-b)!}$, and the posterior probability of D_i under heterozygosity can be computed as:

$$\begin{aligned} &P\left(i\ is\ heterozygosity|D_i = \{X_1, X_2\}\right) \\ &= P\left(D_i = \{X_1, X_2\}|i\ is\ heterozygosity\right) \times P\left(i\ is\ heterozygosity\right) \\ &= C_{RD_i}^{r_1} \times {p_5}^{r_1} \times (1-p_5)^{RD_i - r_1} \times C_{RD_i - r_1}^{r_2} \times {p_5}^{r_2} \times (1-p_5)^{RD_i - r_1 - r_2} \times p_4 \end{aligned} \tag{2}$$

where p_5=0.5, which is the prior probability of heterozygosity, this is because the source of heterozygosity is half of parents. After calculating the two posterior probabilities, the result of judgment is corresponding to the larger value.

Similarly, there is a judgment with the same principle for $\boldsymbol{S}$ when ds=2, just replace p_1 with p_2. Therefore, the posterior probabilities can be calculated as:

$$\begin{aligned} &P\left(i\ is\ homozygosity|D_i = \{x_1, x_2\}\right) \\ &= P\left(D_i = \{x_1, x_2\}|i\ is\ homozygosity\right) \times P\left(i\ is\ homozygosity\right) \\ &= C_{rd_i}^{r_1} \times (1-p_2)^{r_1} \times {p_2}^{rd_i - r_1} \times C_{rd_i - r_1}^{r_2} \times {p_2}^{r_2} \times (1-p_2)^{rd_i - r_1 - r_2} \times p_3 \end{aligned} \tag{3}$$

$$\begin{aligned} &P\left(i\ is\ heterozygosity|D_i = \{x_1, x_2\}\right) \\ &= P\left(D_i = \{x_1, x_2\}|i\ is\ heterozygosity\right) \times P\left(i\ is\ heterozygosity\right) \\ &= C_{rd_i}^{r_1} \times {p_5}^{r_1} \times (1-p_5)^{rd_i - r_1} \times C_{rd_i - r_1}^{r_2} \times {p_5}^{r_2} \times (1-p_5)^{rd_i - r_1 - r_2} \times p_4 \end{aligned} \tag{4}$$

Case 3:

If dl=3, thus $r_1 + r_2 + r_3 = RD_i$, then the posterior probabilities of homozygosity and heterozygosity are given below:

$$
\begin{aligned}
&P\left(i\ is\ homozygosity|D_i=\{X_1,X_2,X_3\}\right)\\
&=P\left(D_i=\{X_1,X_2,X_3\}|i\ is\ homozygosity\right)\times P\left(i\ is\ homozygosity\right)\\
&=C_{RD_i}^{r_1}\times(1-p_1)^{r_1}\times {p_1}^{RD_i-r_1}\times C_{RD_i-r_1}^{r_2}\times\left(\frac{p_1}{2}\right)^{r_2}\times\left(1-\frac{p_1}{2}\right)^{RD_i-r_1-r_2}\times C_{RD_i-r_1-r_2}^{r_3}\times\\
&\left(\frac{p_1}{2}\right)^{r_3}\times\left(1-\frac{p_1}{2}\right)^{RD_i-r_1-r_2-r_3}\times p_3
\end{aligned}\tag{5}
$$

$$
\begin{aligned}
&P\left(i\ is\ heterozygosity|D_i=\{X_1,X_2,X_3\}\right)\\
&=P\left(D_i=\{X_1,X_2,X_3\}|i\ is\ heterozygosity\right)\times P\left(i\ is\ heterozygosity\right)\\
&=C_{RD_i}^{r_1}\times\left(\frac{1-p_1}{2}\right)^{r_1}\times\left(1-\frac{1-p_1}{2}\right)^{RD_i-r_1}\times C_{RD_i-r_1}^{r_2}\times\left(\frac{1-p_1}{2}\right)^{r_2}\times\left(1-\frac{1-p_1}{2}\right)^{RD_i-r_1-r_2}\times\\
&C_{RD_i-r_1-r_2}^{r_3}\times {p_1}^{r_3}\times(1-p_1)^{RD_i-r_1-r_2-r_3}\times p_4
\end{aligned}\tag{6}
$$

It can be seen from equation (15) that the case of homozygosity contains three binomial distributions, namely, the base with the largest proportion is the homozygous allele, whose prior probability is 1-p_1; the remaining bases constitute the noise, the prior probability of the noise is evenly divided. From equation (16), the top two frequency bases are the heterozygous alleles, which divide the prior probability 1-p_1 equally; the rest is the noise, and the prior probability is p_1.

For $\boldsymbol{S}$, if ds=3, there is a similar judgment principle, just replace p_1 with p_2, which is expressed as follows:

$$
\begin{aligned}
&P\left(i\ is\ homozygosity|D_i=\{x_1,x_2,x_3\}\right)\\
&=P\left(D_i=\{x_1,x_2,x_3\}|i\ is\ homozygosity\right)\times P\left(i\ is\ homozygosity\right)\\
&=C_{rd_i}^{r_1}\times(1-p_2)^{r_1}\times {p_2}^{rd_i-r_1}\times C_{rd_i-r_1}^{r_2}\times\left(\frac{p_2}{2}\right)^{r_2}\times\left(1-\frac{p_2}{2}\right)^{rd_i-r_1-r_2}\times C_{rd_i-r_1-r_2}^{r_3}\times\left(\frac{p_2}{2}\right)^{r_3}\times\\
&\left(1-\frac{p_2}{2}\right)^{rd_i-r_1-r_2-r_3}\times p_3
\end{aligned}\tag{7}
$$

$$
\begin{aligned}
&P\left(i\ is\ heterozygosity|D_i=\{x_1,x_2,x_3\}\right)\\
&=P\left(D_i=\{x_1,x_2,x_3\}|i\ is\ heterozygosity\right)\times P\left(i\ is\ heterozygosity\right)\\
&=C_{rd_i}^{r_1}\times\left(\frac{1-p_2}{2}\right)^{r_1}\times\left(1-\frac{1-p_2}{2}\right)^{rd_i-r_1}\times C_{rd_i-r_1}^{r_2}\times\left(\frac{1-p_2}{2}\right)^{r_2}\times\left(1-\frac{1-p_2}{2}\right)^{rd_i-r_1-r_2}\times\\
&C_{rd_i-r_1-r_2}^{r_3}\times {p_2}^{r_3}\times(1-p_2)^{rd_i-r_1-r_2-r_3}\times p_4
\end{aligned}\tag{8}
$$

Similar to the case with d=2, we take the larger value in equations (15) and (16) as the judgment result of $\boldsymbol{L}$. Equations (17) and (18) correspond to $\boldsymbol{S}$.

Case 4:

If dl=4, thus $r_1+r_2+r_3+r_4=RD_i$, then the posterior probabilities of homozygosity and heterozygosity are given below:

$$
\begin{aligned}
&P\left(i\ is\ homozygosity|D_i=\{X_1,X_2,X_3,X_4\}\right)\\
&=P\left(D_i=\{X_1,X_2,X_3,X_4\}|i\ is\ homozygosity\right)\times P\left(i\ is\ homozygosity\right)\\
&=C_{RD_i}^{r_1}\times(1-p_1)^{r_1}\times {p_1}^{RD_i-r_1}\times C_{RD_i-r_1}^{r_2}\times\left(\frac{p_1}{3}\right)^{r_2}\times\left(1-\frac{p_1}{3}\right)^{RD_i-r_1-r_2}\times C_{RD_i-r_1-r_2}^{r_3}\times\left(\frac{p_1}{3}\right)^{r_3}\times\\
&\left(1-\frac{p_1}{3}\right)^{RD_i-r_1-r_2-r_3}\times C_{RD_i-r_1-r_2-r_3}^{r_4}\times\left(\frac{p_1}{3}\right)^{r_4}\times\left(1-\frac{p_1}{3}\right)^{RD_i-r_1-r_2-r_3-r_4}\times p_3
\end{aligned}
\tag{9}
$$

$$
\begin{aligned}
&P\left(i\ is\ heterozygosity|D_i=\{X_1,X_2,X_3,X_4\}\right)\\
&=P\left(D_i=\{X_1,X_2,X_3,X_4\}|i\ is\ heterozygosity\right)\times P\left(i\ is\ heterozygosity\right)\\
&=C_{RD_i}^{r_1}\times\left(\frac{1-p_1}{2}\right)^{r_1}\times\left(1-\frac{1-p_1}{2}\right)^{RD_i-r_1}\times C_{RD_i-r_1}^{r_2}\times\left(\frac{1-p_1}{2}\right)^{r_2}\times\left(1-\frac{1-p_1}{2}\right)^{RD_i-r_1-r_2}\times\\
&C_{RD_i-r_1-r_2}^{r_3}\times\left(\frac{p_1}{2}\right)^{r_3}\times\left(1-\frac{p_1}{2}\right)^{RD_i-r_1-r_2-r_3}\times C_{RD_i-r_1-r_2-r_3}^{r_4}\times\left(\frac{p_1}{2}\right)^{r_4}\times\left(1-\frac{p_1}{2}\right)^{RD_i-r_1-r_2-r_3-r_4}\times p_4
\end{aligned}
\tag{10}
$$

For $\boldsymbol{S}$, if ds=4, the judgment principle is expressed as follows:

$$
\begin{aligned}
&P\left(i\ is\ homozygosity|D_i=\{x_1,x_2,x_3,x_4\}\right)\\
&=P\left(D_i=\{x_1,x_2,x_3,x_4\}|i\ is\ homozygosity\right)\times P\left(i\ is\ homozygosity\right)\\
&=C_{rd_i}^{r_1}\times(1-p_2)^{r_1}\times {p_2}^{rd_i-r_1}\times C_{rd_i-r_1}^{r_2}\times\left(\frac{p_2}{3}\right)^{r_2}\times\left(1-\frac{p_2}{3}\right)^{rd_i-r_1-r_2}\times C_{rd_i-r_1-r_2}^{r_3}\times\left(\frac{p_2}{3}\right)^{r_3}\times\\
&\left(1-\frac{p_2}{3}\right)^{rd_i-r_1-r_2-r_3}\times C_{rd_i-r_1-r_2-r_3}^{r_4}\times\left(\frac{p_2}{3}\right)^{r_4}\times\left(1-\frac{p_2}{3}\right)^{rd_i-r_1-r_2-r_3-r_4}\times p_3
\end{aligned}
\tag{11}
$$

$$
\begin{aligned}
&P\left(i\ is\ heterozygosity|D_i=\{x_1,x_2,x_3,x_4\}\right)\\
&=P\left(D_i=\{x_1,x_2,x_3,x_4\}|i\ is\ heterozygosity\right)\times P\left(i\ is\ heterozygosity\right)\\
&=C_{rd_i}^{r_1}\times\left(\frac{1-p_2}{2}\right)^{r_1}\times\left(1-\frac{1-p_2}{2}\right)^{rd_i-r_1}\times C_{rd_i-r_1}^{r_2}\times\left(\frac{1-p_2}{2}\right)^{r_2}\times\left(1-\frac{1-p_2}{2}\right)^{rd_i-r_1-r_2}\times C_{rd_i-r_1-r_2}^{r_3}\times\\
&\left(\frac{p_2}{2}\right)^{r_3}\times\left(1-\frac{p_2}{2}\right)^{rd_i-r_1-r_2-r_3}\times C_{rd_i-r_1-r_2-r_3}^{r_4}\times\left(\frac{p_2}{2}\right)^{r_4}\times\left(1-\frac{p_2}{2}\right)^{rd_i-r_1-r_2-r_3-r_4}\times p_4
\end{aligned}
\tag{12}
$$

Case 5:

If dl=5, thus $r_1+r_2+r_3+r_4+r_5=RD_i$, then the posterior probabilities of homozygosity and heterozygosity are given below:

$$
\begin{aligned}
&P\left(i\ is\ homozygosity|D_i=\{X_1,X_2,X_3,X_4,X_5\}\right)\\
&=P\left(D_i=\{X_1,X_2,X_3,X_4,X_5\}|i\ is\ homozygosity\right)\times P\left(i\ is\ homozygosity\right)\\
&=C_{RD_i}^{r_1}\times(1-p_1)^{r_1}\times {p_1}^{RD_i-r_1}\times C_{RD_i-r_1}^{r_2}\times\left(\frac{p_1}{4}\right)^{r_2}\times\left(1-\frac{p_1}{4}\right)^{RD_i-r_1-r_2}\times C_{RD_i-r_1-r_2}^{r_3}\times\left(\frac{p_1}{4}\right)^{r_3}\times\\
&\left(1-\frac{p_1}{4}\right)^{RD_i-r_1-r_2-r_3}\times C_{RD_i-r_1-r_2-r_3}^{r_4}\times\left(\frac{p_1}{4}\right)^{r_4}\times\left(1-\frac{p_1}{4}\right)^{RD_i-r_1-r_2-r_3-r_4}\times C_{RD_i-r_1-r_2-r_3-r_4}^{r_5}\times\\
&\left(\frac{p_1}{4}\right)^{r_5}\times\left(1-\frac{p_1}{4}\right)^{RD_i-r_1-r_2-r_3-r_4-r_5}\times p_3
\end{aligned}
\tag{13}
$$

$$
\begin{aligned}
&P\left(i \text{ } is \text{ } heterozygosity|D_i=\{X_1,X_2,X_3,X_4,X_5\}\right)\\
&=P\left(D_i=\{X_1,X_2,X_3,X_4,X_5\}|i \text{ } is \text{ } heterozygosity\right)\times P\left(i \text{ } is \text{ } heterozygosity\right)\\
&=C_{RD_i}^{r_1}\times\left(\frac{1-p_1}{2}\right)^{r_1}\times\left(1-\frac{1-p_1}{2}\right)^{RD_i-r_1}\times C_{RD_i-r_1}^{r_2}\times\left(\frac{1-p_1}{2}\right)^{r_2}\times\left(1-\frac{1-p_1}{2}\right)^{RD_i-r_1-r_2}\times\\
&C_{RD_i-r_1-r_2}^{r_3}\times\left(\frac{p_1}{3}\right)^{r_3}\times\left(1-\frac{p_1}{3}\right)^{RD_i-r_1-r_2-r_3}\times C_{RD_i-r_1-r_2-r_3}^{r_4}\times\left(\frac{p_1}{3}\right)^{r_4}\times\left(1-\frac{p_1}{3}\right)^{RD_i-r_1-r_2-r_3-r_4}\times\\
&C_{RD_i-r_1-r_2-r_3-r_4}^{r_5}\times\left(\frac{p_1}{3}\right)^{r_5}\times\left(1-\frac{p_1}{3}\right)^{RD_i-r_1-r_2-r_3-r_4-r_5}\times p_4 \qquad (14)
\end{aligned}
$$

For $\boldsymbol{S}$, if ds=5, the judgment principle is expressed as follows:

$$
\begin{aligned}
&P\left(i \text{ } is \text{ } homozygosity|D_i=\{x_1,x_2,x_3,x_4,x_5\}\right)\\
&=P\left(D_i=\{x_1,x_2,x_3,x_4,x_5\}|i \text{ } is \text{ } homozygosity\right)\times P\left(i \text{ } is \text{ } homozygosity\right)\\
&=C_{rd_i}^{r_1}\times(1-p_2)^{r_1}\times {p_2}^{rd_i-r_1}\times C_{rd_i-r_1}^{r_2}\times\left(\frac{p_2}{4}\right)^{r_2}\times\left(1-\frac{p_2}{4}\right)^{rd_i-r_1-r_2}\times C_{rd_i-r_1-r_2}^{r_3}\times\left(\frac{p_2}{4}\right)^{r_3}\times\\
&\left(1-\frac{p_2}{4}\right)^{rd_i-r_1-r_2-r_3}\times C_{rd_i-r_1-r_2-r_3}^{r_4}\times\left(\frac{p_2}{4}\right)^{r_4}\times\left(1-\frac{p_2}{4}\right)^{rd_i-r_1-r_2-r_3-r_4}\times C_{rd_i-r_1-r_2-r_3-r_4}^{r_5}\times\\
&\left(\frac{p_2}{4}\right)^{r_5}\times\left(1-\frac{p_2}{4}\right)^{rd_i-r_1-r_2-r_3-r_4-r_5}\times p_3 \qquad (15)
\end{aligned}
$$

$$
\begin{aligned}
&P\left(i \text{ } is \text{ } heterozygosity|D_i=\{x_1,x_2,x_3,x_4,x_5\}\right)\\
&=P\left(D_i=\{x_1,x_2,x_3,x_4,x_5\}|i \text{ } is \text{ } heterozygosity\right)\times P\left(i \text{ } is \text{ } heterozygosity\right)\\
&=C_{rd_i}^{r_1}\times\left(\frac{1-p_2}{2}\right)^{r_1}\times\left(1-\frac{1-p_2}{2}\right)^{rd_i-r_1}\times C_{rd_i-r_1}^{r_2}\times\left(\frac{1-p_2}{2}\right)^{r_2}\times\left(1-\frac{1-p_2}{2}\right)^{rd_i-r_1-r_2}\times C_{rd_i-r_1-r_2}^{r_3}\times\\
&\left(\frac{p_2}{3}\right)^{r_3}\times\left(1-\frac{p_2}{3}\right)^{rd_i-r_1-r_2-r_3}\times C_{rd_i-r_1-r_2-r_3}^{r_4}\times\left(\frac{p_2}{3}\right)^{r_4}\times\left(1-\frac{p_2}{3}\right)^{rd_i-r_1-r_2-r_3-r_4}\times C_{rd_i-r_1-r_2-r_3-r_4}^{r_5}\times\\
&\left(\frac{p_2}{3}\right)^{r_5}\times\left(1-\frac{p_2}{3}\right)^{rd_i-r_1-r_2-r_3-r_4-r_5}\times p_4 \qquad (16)
\end{aligned}
$$
